# End-to-end experimentation of a 5G vertical within the scope of blended learning

**DOI:** 10.1007/s43926-021-00008-5

**Published:** 2021-02-24

**Authors:** Ngombo Armando, Rui Almeida, José Marcelo Fernandes, Jorge Sá Silva, Fernando Boavida

**Affiliations:** 1 Centre for Informatics and Systems of the University of Coimbra (CISUC), 3030-290 Coimbra, Portugal; 2 Instituto Politécnico, Universidade Kimpa Vita, Nkondo Mbenza 077, Uíge, Angola; 3 Department of Informatic Engineering (DEI), University of Coimbra, 3030-290 Coimbra, Portugal; 4 Institute for Systems Engineering and Computers at Coimbra (INESC Coimbra), 3030-290 Coimbra, Portugal; 5 Department of Electrical and Computer Engineering (DEEC), University of Coimbra, 3030-290 Coimbra, Portugal

**Keywords:** 5G, Blended learning, FIWARE

## Abstract

The next generation of mobile networks, widely known as 5G, was designed to respond to the next 10 years’ communication challenges. 5G will be an essential structure on which all types of electronic communications will rely. Built on the emerging 5G technology, we developed a vertical solution for blended learning environments within a Portuguese national project scope. The project aims to propose an integrated demonstration of a set of products capable of being part and providing services in the future 5G networks framework. To this end, we adopted a bottom-up approach to design and develop a product named *5GOpenclasses*, where we leveraged FIWARE middleware to manage all entities. This paper presents the architecture, technological platform, associated data structures, and end-user applications of *5GOpenclasses*. We also present the design of innovative location-based service for blended learning environments. This paper is the first step of the proposed product towards its quantitative evaluation when running Over-the-Top on both 4G and 5G networks. Although successful unit tests were carried out in what concerns the functional outcome, the integration tests for quantitative results depend on the availability of other project components.

## Introduction

The defined use cases for 5G are high-level expectations that drive the development of all components for the communications infrastructure in 2020 and beyond. Such use-cases are known as enhanced Mobile Broadband (eMBB), massive Machine Type Communications (mMTC) and ultra-reliable and Low-Latency Communications (urLLC) [1]. In this context, we designed and developed a product named *5GOpenclasses* to support blended learning activities.

*5GOpenclasses* includes a mobile application that allows students to view high definition (HD) live streams of the class sessions, access materials from a multimedia repository and use a real-time chat. During the class sessions, remote students can interact with teachers. A teacher can create and delete classrooms from a web application interface and upload, edit, or delete files from the backend application server. An array of live video streams serving several students is an ideal scenario to leverage the eMBB network capability, while the collection of both mobile and stationary sensor data enables to assess the network's mMTC capability. The reactivity on providing the students' tailored resources, depending on their location, is a relevant test case for the urLLC capability.

In this paper, we present the approach that we adopted towards the implementation of a 5G-ready product. We highlight the design methodology that is based on a bottom-up approach and good practices from the literature for successful development of blended learning environments in 5G. We also provide a demonstration video clip with the product's functional results and present the design for the quantitative assessment of the test cases, when running Over-the-Top (OTT), on both 4G and 5G 3GPP networks.

The remainder of this paper is organised as follows. In Sect. 2, we present the related work and the context of the project. In Sect. 3, we describe the architecture and the components of the proposed solution. Section 4 focuses on end-user applications of *5GOpenclasses*. In Sect. 5, we present the unit tests carried out regarding the functional outcome, and we address the scenario designed for the integrated demonstration. Finally, we provide concluding remarks in Sect. 6.

## Related work and context

### Related work

The effective design of blended learning environments should consider four aspects: incorporate flexibility, stimulate interaction, facilitate student learning processes, and foster a productive learning environment [2]. Information and Communication Technologies as a support tool for better education is not new and is not meant to replace face-to-face activities. Amid pandemic situations such as COVID-19, online teaching facilities and blended learning environments are essential to avoid the lockdown of training programs. The exercise of designing a blended learning environment must be a joint work by both researchers and practitioners on education.

According to Boelens et al*.* [2], such design must incorporate solutions that target the following question: which amount of flexibility is desirable? As for the stimulating interaction, it is interesting to mention that many learners do not want to lose the social interaction and human touch they are used to in a face-to-face environment while benefiting from the blended learning method's flexibility. There exist different categories of effective strategies to foster a productive learning environment. The strategies include motivating, concentrating and exerting efforts and attributing and judging oneself, appraising, and finally, dealing with emotions. Overall, the comprehensive literature review by Boelens et al*.* shows that the implementation of a blended learning environment must rely on a strategy from the institution that wants to adopt it. The institution should also choose activities that can be blended and introduce a new culture for all the stakeholders.

Recent studies have been presented with approaches to leverage both 5G and the Internet of Things (IoT) into blended learning environments. In [3], Ever and Rajan investigated and identified how these emerging technologies would be integrated into education sciences like medicine. Their findings include the fact that 5G will foster mobile learning by providing educators with efficient learning scenarios that satisfy high priority learner requirements. To Ever and Rajan, security and privacy are also the focus in developing future solutions on blended learning environments over 5G.

In [4], Zhongmei et al. measured the effectiveness of learning through long-distance digital support. They submitted a Quality of Experience (QoE) questionnaire to 180 college students through a Mobile Communication course, comprising both theoretical and practical operation exercises. The authors recommended that schools try to integrate both 5G and IoT technologies to their hybrid teaching in the future. To them, 5G will shorten the download delay time and expand the capacity of mixed reality content and video of the class sessions. An *automatic login when entering the classroom* is an example of leveraging IoT to enhance both learning and teaching experience. Likewise, the possibility of gathering sensor data would be exploited to analyse any relevant information in real-time to help teachers successfully deliver lectures.

Similarly, Baratè et al*.* [5] presented an example of the adoption of 5G technologies in an educational context. More specifically, they presented an application to music education laboratory activities based on Augmented Reality (AR) and Virtual Reality (VR). The authors state that teaching music to remote students is problematic because it implies the exchange of high-quality audio streams and symbolic information such as score, metadata, and lyrics. The complexity in remote teaching of music increases when combined with AR/VR format classes. For this reason, the authors believe that available communication technologies are not suitable for experiencing this kind of didactic activities. Hence, the capabilities envisioned in 5G would be relevant to remote live music classes’ needs.

The studies presented above give essential guidance for designing an effective blended learning environment [2, 3], over 5G [3–5] and IoT technologies [3, 4]. The implemented solutions in [3–5] are described as based on a top-down approach. Accordingly, we believe that their proposed designs are more generic to the supported communication network and thus; they risk not to produce fully optimised products for the 5G network as envisioned in [1].

Based on the guidelines from [2–5], we designed and developed a product named *5GOpenclasses*. Our design relies on a bottom-up approach; that is, we started by studying the capabilities offered by the future 5G network, and then set a solution that makes the most of it. The main advantage of a bottom-up approach is that a proposed artefact is designed based on the reality of the network enablers. Thus, the solution is thought to offer a fully optimized product for 5G network. The first novelty of the current work is the exploitation, in a single product, of all use-cases described in the 5G vision [1]. Second, we propose a process to provide a differentiated service on-the-fly, according to the couple UE's location. Next, we will present the context of our study and the proposed solution.

### Context of the project

We developed a use case study within the scope of a national project named *Mobilizador 5G* (https://5go.pt/en/home/). For Portugal, this is one of the main projects to provide 5G Processes, Products and Services. From a technological viewpoint, the project aimed to research, develop, validate, integrate, and demonstrate both mechanisms and solutions to provide services in the framework of future 5G networks. The project also aims to join and harmonise different telecommunication operators' efforts, intending to create innovative solutions for markets in both Business-to-Business and Business-to-Customer models. The targeted products should cover all the functional domains in 5G networks, namely access network, core network, and vertical sectors organised into machine-to-machine communication and human communication.

*Mobilizador 5G* involved fourteen companies, research, and innovation centres under Altice Labs' leadership—Portugal. The set of Processes, Products and Services to be developed by the project partners are expected to be of Technology Readiness Level (TRL) 7/8 [6] and will be assessed in an integrated demonstration infrastructure.

In this context, we proposed a product for human communication that would stress the supported communication network concerning its capabilities for latency, broadband and machine-type communications. To this end, we designed, developed, and tested a product to support blended learning activities named *5GOpenclasses*.

## Proposed solution

### Architecture

**Fig. 1:**
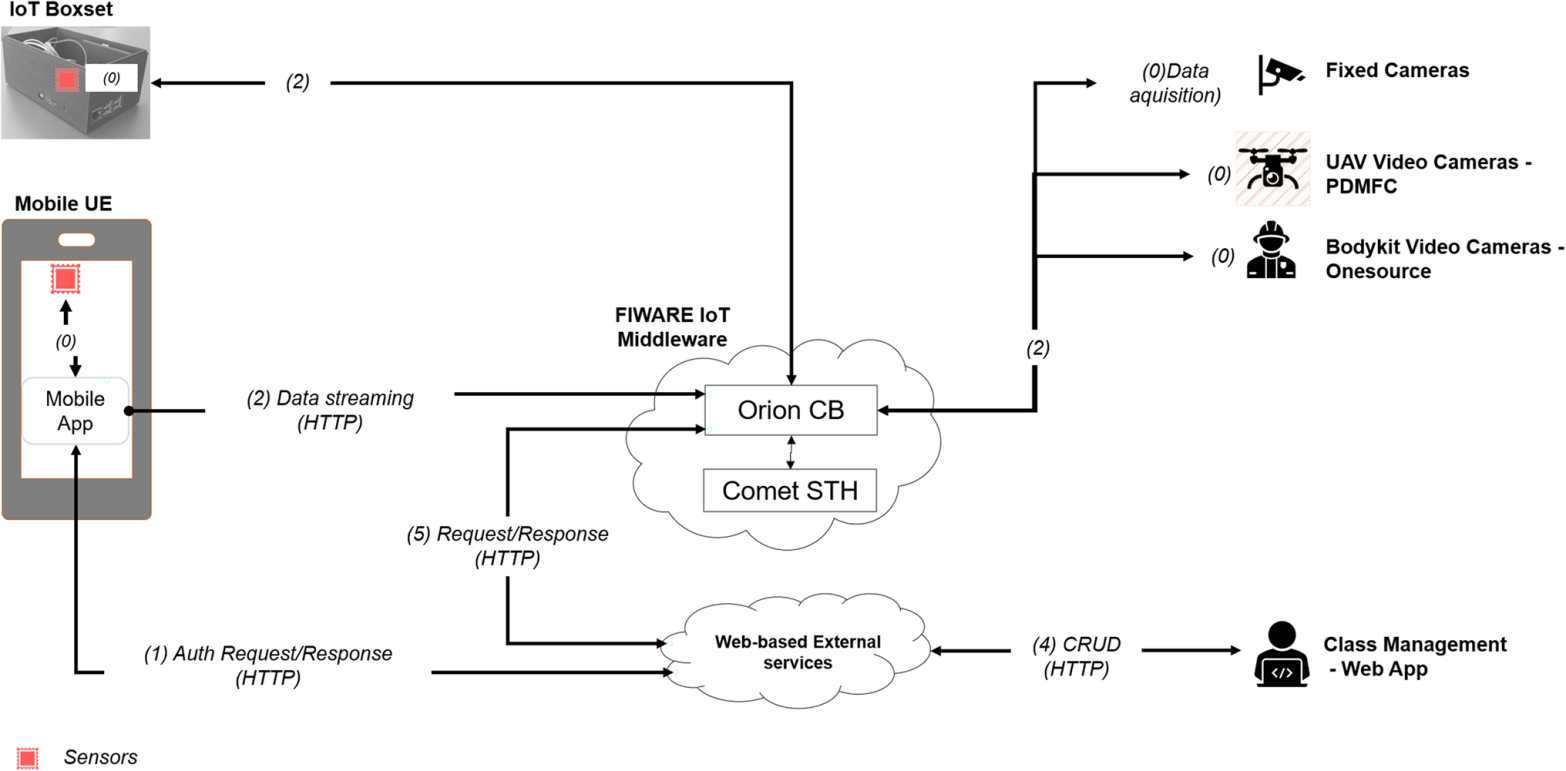
5GOpenclasses—Technologies and data flows

The architecture of the *5GOpenclasses* is displayed in Fig. 1, which we will explain the different components. The IoT boxset is a 3D printed component that we developed in the scope of a previous project [7], to monitor the temperature, light and noise of the surroundings, in this case, the physical classroom. The IoT boxset contains a Raspberry Pi gateway, an Arduino board, and sensors for communication, preprocessing, and sensing purposes, respectively. The mobile User Equipment (UE) is an Android OS handheld device that runs the *5GOpenclasses* mobile application serving the students to view live streams of the class sessions, replay and download videos from a repository, and chat with both teachers and classmates. More details about the mobile application will be given in Sect. 4.1.

We can collect two categories of sensor data during the class sessions, namely, mobile and stationary. The former comprises gyroscope, accelerometer, proximity, sound, and light. The latter comprises the ones from the IoT boxset. Overall, the collected data from sensors feed several analytical services so that the teachers can be able to monitor the influence of the sensed values in the students' performances as we have also exploited in previous works [7, 8].

The core functions of the *5GOpenclasses* system are represented in the middle of Fig. 1. The application server is based on FIWARE IoT middleware that will be described in Sect. 3.2. The application server handles the provision of session control and media server. The application server also hosts the storage for the class sessions materials.

The Class Management component is provided by a web application that serves both the teachers and the administrator of the 5GOpenclasses system. Via this application, a teacher can create, manage and close class sessions. In turn, the administrator can, for instance, add new subjects to teachers or view the current list of classes per subject. More details about the web application will be given in Sect. 4.2.

At the top-right side of Fig. 1, we can see different sources of videos streams. During the class session, students can consume an array of live video both from indoors (fixed cameras) and outdoors (attached to Unmanned Aerial Vehicles (UAV) 360° cameras and cameras attached to firefighters suits labelled *Bodykit)*. Two partners of the project provide the outdoors streams from the two type of cameras. Likewise, the optimised video streaming will rely on an eMBB slice to be configured by the partner of the project in charge of the 3GPP mobile networks.

**Fig. 2:**
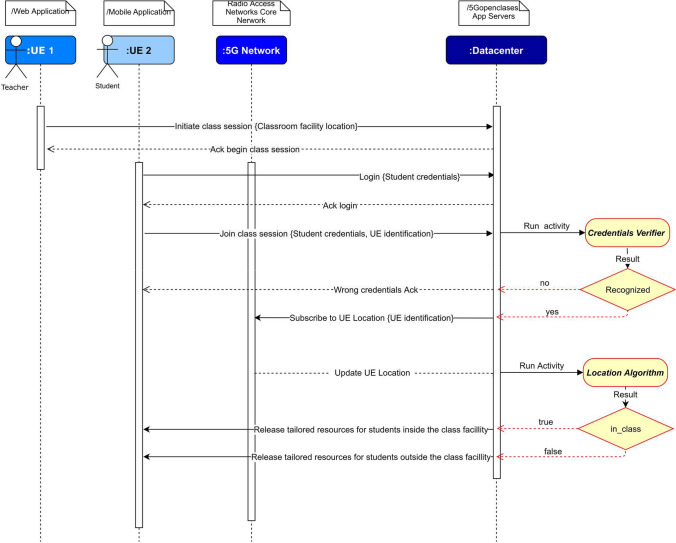
Location-based service sequence diagram

In *5GOpenclasses*, we propose that the students not be able to chat, ask questions, and watch the live streams if they were detected inside the physical classroom facility. Moreover, the mobile sensors data are to be collected only for students detected as being inside the physical classroom facility. For the current testbed of the *5GOpenclasses*, this detection is time-based. Hence, every 30 s, we toggle the value of a Boolean named *in_class* (see Table 1), an attribute of the *Student* entity. However, in an advanced version of the *5GOpenclasses*, the *in_class* values are to be managed automatically by the process that is depicted in Fig. 2.

**Table 1:** 5Gopenclasses—data structure

Entities	Attributes
Users	id, type, name, email, password, is_online, in_class, TimeInstant, gyroscope, accelerometer, proximity, sound, light, create_time, update_time
Teachers	id, type, name, email, password, create_time, update_time
Rooms	id, type, password, name, description, is_online, recording, prof_id, course_id, create_time, update_time, location
Courses	id, type, academic_year, profs, create_time, update_time
Questions	id, type, asking, room_id, user_id, create_time, update_time
Group_Rooms	id, type, creator_id, password, create_time, update_time
Ratings	id, type, comment, rate, recommend, room_id, create_time, update_time
Media_Files	id, type, download_path, file_name, room_id, create_time, update_time
Environment	id, type, TimeInstant, light, noise, temperature

The automatic location-based service was not developed in the initial version of *5GOpenclasses* because it involves interfaces from different consortium stakeholders that must be carefully articulated. Furthermore, the privacy issue related to the UE tracking that must be carefully considered. The sequence diagram for the location-based service in Fig. 2 works as follows.

Firstly, the teacher initiates the class session and automatically informs the classroom session's location to the application server (*location* Attribute from *Rooms* entity in Table 1). The location can be based on Global Navigation Satellite System (GNSS), plus the elevation coordinates or any other format compliant with the mobile network location data type. The location may include the coordinates of a single point of the classroom or an array of coordinates that enables the representation of the entire classroom object in the space. When a student logs in and joins a class session, the mobile application will send the users credentials and its UE identification to the application server.

The UE identification can be any unique number that enables the mobile network to track its location throughout the cells. E.g., the International Mobile Equipment Identity (IMEI), the Integrated Circuit Card Identifier (ICCID), and the International Mobile Subscriber Identity (IMSI). The user credentials serve to authenticate the users in the *5GOpenclasses* application server. If the credentials are recognised, the application server will send the associated UE identification to the mobile network entities in charge of tracking the UEs. The mobile network must provide the application server with the initial UE location and the updated values. Such communication is to be made via a subscription so that whenever UE moves, the mobile network publishes the new location coordinates to the application server.

Both the initial and updated locations coordinates of the UE feed a computational process tagged as *Location Algorithm* in Fig. 2. This algorithm will compare the pre-registered location of the *Room* entity with the current location of the UE. If the point UE is located inside the object *Room,* then the *in_class* value will be *true*. Else, *the in_class* value will be *false*. Finally, the mobile application will release tailored resources to the end-users, depending on its binary location. As we proposed for the current version of the product, if *in_class* = *true*, the mobile application will deactivate the chat, ask questions functionality, and live streaming. With *in_class* = *false,* the mobile sensors data will not be collected.

### Core servers

#### FIWARE platform

FIWARE is a European set of specifications to enable the smooth development of smart applications in multiple vertical sectors. FIWARE is open, public and royalty-free. The FIWARE project started in 2011 within the Seventh Framework Programme (FP7) of the European Commission as part of the Future Internet Public–Private Partnership Programme (FI–PPP). The FI–PPP's primary goal was to advance a process for harmonised European technology platforms and their implementation. Moreover, the FI–PPP aim was to provide integration and harmonisation of relevant policies frameworks.

The process to establish FIWARE in the market was divided into three phases. The first phase aimed to create the technological core, while the second mainly aimed at implementing the FIWARE data centers known as *nodes*. The last phase aimed primarily at creating a sustainable ecosystem for Small Medium Enterprises, through the selection of sixteen business accelerators.

**Fig. 3:**
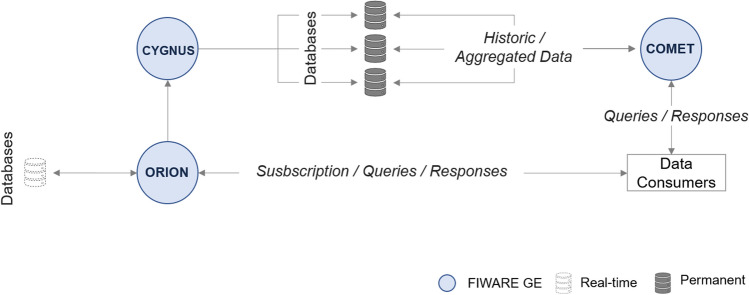
Typical components of FIWARE platform

FIWARE is based on a library of components called Generic Enablers (GEs) that offer reusable and commonly shared functions as a Service exposed by RESTful APIs. Figure 3 portrays the typical components of the FIWARE platform.

The FIWARE project adopts the Next-Generation Service Interface (NGSI) 9/10 data model, previously developed by the Open-Mobile Alliance (OMA) [9]. The model is based on entities and attributes. Functions in *5GOpenclasses* need to be scalable, and dynamic models are needed to represent such a system's data. Each entity has its type and is represented by attributes, using JSON format.

The ORION GE manages all the entities in 5GOpenclasses. Thus, it is in ORION where entities can be created, deleted, retrieved, and updated. This module acts as a broker, by allowing external systems (consumers) to make data subscriptions with specific rules to the entities and attributes, which improves the architectural distribution and scalability. ORION sends notifications to these consumers when subscription rules are observed. The last values of information sent by the entities are stored in the attached ORION databases. However, ORION is not fit for storing historic data. Instead, the Cygnus module is the GE in charge of persisting specific data sources in several storages, using the Apache Flume technology and subscribing to the ORION entities.

Accordingly, when a new entity arrives at the Cygnus GE, the listener will put it in a specific channel and forward it to a third-party data storage (e.g., MySQL, MongoDB and CKAN channel).

Databases usually do not provide APIs to retrieve the data to applications. Therefore, another module of the FIWARE platform handles this issue, namely the STH -Comet GE. STH provides a RESTFUL API with historic-queries capabilities, and aggregated methods so that each time the end applications need to access historic data, the Comet will connect to the MongoDB and retrieve it.

As for the FIWARE implementation, the *5GOpenclasses* application server runs a cloud-based Virtual Machine (VM) with a docker environment. The docker environment serves to manage a suite of containers that implement the GEs.

#### WebRTC video streaming

We are using a Kurento Web Real-Time Communication (WebRTC) server to work as a receiver and transmitter for real-time video streams. Kurento is also a GE of FIWARE. While standard WeRTC media servers' tasks include transcoding, Multipoint Control Unit (MCU) and recording, Kurento server adds tasks such as flexible processing, augmented reality, blending, mixing, and analysing. We recall that the *5GOpenclasses* web application provides the features for recording and transmitting video to students. These video streams are redirected to a WebRTC multimedia server. When the students want to view these contents, they can do so through the mobile application. In this scenario, we leverage a One-to-Many video call real-time transmission architecture.

Due to security reasons, we had to deploy two independent servers, namely a STUN Server [for Session Traversal of User Datagram Protocol (UDP) Through Network Address Translators (NATs)] and a TURN Server (for Traversal Using Relays around NAT. In a real context, most devices are behind firewalls, where NAT rules filter the communications. STUN servers are used to obtain an IP address and an external network port, while TURN servers are used to relay traffic if the direct connection (device to device) fails. In other words, the overall purpose of STUN servers is to obtain external IP/port addresses, and TURN servers are used to relay traffic if the peer-to-peer connection fails.

All devices that want to offer/receive real-time data content must be synchronised temporally to be able to communicate in real-time. This simultaneous synchronisation process is called *signalling* and is used to exchange information of:

- session control,
- error messages,
- transmission channel data
- critical data for establishing secure connections and
- network data (IP address and port).

Even if the devices that should receive content in real-time are synchronised, they should not yet be able to do so because of NATs and firewalls filtering rules. WebRTC applications can use the Impact, Confidence, and Ease (ICE) framework to overcome today’s networks’ complexity. An ICE framework is meant to find the best way to connect devices. To achieve such a task, the best path to connect the devices is tested in the following order of options:

- A direct connection between devices (impossible due to NATs)
- The utilisation of an external address of a STUN server
- If it fails, the traffic is directed through a TURN relay server.

NATs offer the possibility of communicating an internal IP address of a local network with the outside, through its translation. There is no possibility for an IP address in a peer-to-peer scenario on a private network to be used as a public address. To overcome this issue, WebRTC uses STUN servers that are hosted on the public Internet. Besides, WebRTC applications use STUN servers to discover both the end-device public IP address and port, thus enabling the transmission of that public address to the peer of direct transmission (peer-to-peer transmission). The exchange of both the IP and public port between the transmitter and receiver of multimedia content is done through the signalling mechanism. The result of this communication with the STUN servers is the establishment of a direct connection between IP addresses/external ports.

For most WebRTC applications to function normally, it is necessary to use servers to relay traffic between the multimedia content provider and receiver. Indeed, it is often impossible to create a direct connection between them. The most common way to solve this problem is to use a TURN server since it is a network traffic relay protocol that assists the bypass of NATs and firewalls. In other words, a TURN server can be used to relay audio/video/data streams between peer devices.

### Data structure

The structure of the data object storage in *5GOpenclasses* system is as presented in Table 1. All passwords are encrypted in SHA-256 format. The *update_time* attribute values are updated by the host machine’s current date, in ISO 8601 format (e.g., 2020-02-19T13:58:26.00Z). The media files in the *5GOpenclasses* repository contain the path to their downloading and are associated with a past class session. These multimedia files can be stored in different formats, and the paths to download them are indicated in the *download_path* attribute of the *Media_Files* entity.

## End-user applications

### Mobile application

We are currently using *webrtcpeer-Android* library to support WebRTC communications. As described in Sect. 3.2, the mobile application only receives real-time video content from the teacher since the communication architecture with the Kurento multimedia server is a One-to-Many video call. Therefore, we can identify that the stream comes only from one teacher, while there can be several students.

The communication of this video stream is of a one-way type, i.e., the stream goes from the source of the video (web application) to the student's video destination (mobile application). Besides the indoor cameras' video streams, *5GOpenclasses* provides video stream contents from both UAV and *Bodykit* outdoors cameras, via a Janus WebRTC server. Janus is a server that implements extra functionalities that go beyond the features that we initially designed for WebRTC real-time communications. In the mobile application case, we implemented a classroom dedicated to the cameras where the video streams are received and presented.

For the QoE assessment purposes, students are invited to answer an electronic form that appears in the mobile application before they leave a class session. The form comprises two mandatory rating questions and a free-comment space. The data collected from the user's feedback are sent to the application server, more precisely, to the *Ratings* Entity in ORION. Both rates and comments are to feed an external service that performs Mean Opinion Scores (MOS) [10] and Topic modelling [11].

The registration/authentication process leverage an external service implemented by a project partner (IT Aveiro). This service is based on the Privacy-Preserving User Authentication Protocol (PPUAP) described in [12]. This process corresponds to the *Credentials Verifier* in Fig. 2.

### Web application

We developed the web application in Python using the Dash by *Plotly* framework. The Dash by *Plotly* framework is an open-source framework dedicated to building data analytics web applications based on Flask, Plotly.js, and React.js.

The construction of this end-user application relies on using a WebRTC Kurento server, supported by STUN and TURN servers. Consequently, we can stream video content in real-time leveraging the One-to-Many video calls architecture. We chose this server architecture because our target scenario is providing tools that enable the teacher to transmit their classes online and in real-time to their students.

For real-time video streaming purposes, a teacher can broadcast its computer screen contents such as slide presentations and his webcam. Finally, a teacher can manage its class sessions, share complementary materials s, view the list the subjects assigned to him and answer the questions asked by students during class sessions. The data generated for these activities are stored on-the-fly, on the central databases.

## Testes functionalities and assessment design

We have successfully tested the main functionalities of the *5GOpenclasses* listed in Table 2, within a LAN environment. The multimedia results namely a video clip and the pertained screenshots, are available online at https://bit.ly/393FR5c.

**Table 2:** Tested functionalities

#	Tested functionalities	Comments
1	Secure Signup and Sign in for the mobile application	Via PPUAP from IT Aveiro
2	Room creation	As soon as the class session is created, students may securely log to it
3	Starting a class session	This functionality is the one that opens the full panel for a teacher to manage a class session, e.g., see the asked questions or its own stream feed
4	Student registration and authenticated login	This is the only way students can access the functionalities of the mobile application
5	Authenticated access to classes and video streams reception	The student can select different views from the array of available streams of a class session
6	Interventions to classes	All students attending the class session can visualize the intervention of the colleagues
7	Management of the repository by the teacher (feeding, renaming, suppression)	The repository supports different digital formats (e.g..doc,.ppt.,.avi,.mp3)
8	Resource consumption du repository	The available materials can be downloaded or played directly from the repository
9	QoE questionnaire triggering and answering	The questionnaire is triggered at the end of each class session
10	Questionnaire data collection by the server	The results of the questionnaires are sent to the backend App server (FIWARE—Orion)
11	Integrated Video sources	Video and audio from Drones and Bodykit. Ready for the integrated demonstration
12	Collect and display mobile sensor data	The UE must be provided with the required sensors hardware
13	Collect and display stationary sensor data	IoT Boxset developed by the University of Coimbra

**Fig. 4:**
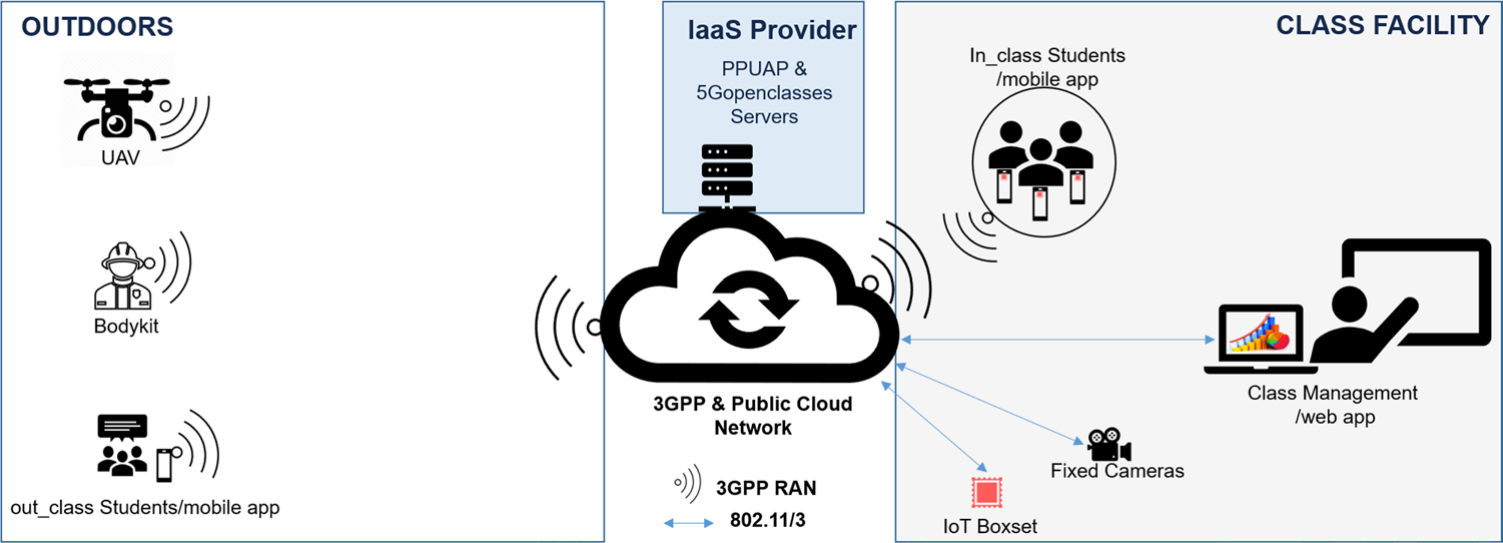
Integrated demonstration scenario

The product’s final assessment is to be carried out in an integrated demonstration scenario displayed in Fig. 4, which involves all the partners in the national project consortium. It is important to remind that the components of Fig. 4 are those already described in Sects. 3 and 4.

Table 3 shows the designed test plan for the integrated scenario where the main goal will be to compare the product's behaviour when running the test cases OTT on both 4G to 5G networks simultaneously. We will split the tests into three types, namely:

- Integration with other partners of the project;
- Validation of the *5GOpenclasses* functionalities in a production-like scenario;
- Evaluation of the product from QoS viewpoint.

**Table 3:** Run tests and indicators

#	Test cases	Test type^a^	QoS indicators
1	From the web application: creation of a room with 3 video streams (fixed camera, UAV and bodykit)	i, ii	Not applicable (Fully LAN-based communication)
2	From the mobile application: registration via PPUAP	i, ii, iii	Latency
3	From the mobile application: access to a room and consumption of video streaming	i, ii, iii	Latency, Jitter, Loss, Bit Rate & Play Rate, filling in The Buffer, Post-Buffer Lag Length, Play Length of Video, Lag Ratio of Video
4	From the web application: upload an HD video to the repository	ii, iii	Not applicable (Fully LAN-based communication)
5	From the mobile application: direct consumption of the video from the repository	ii, iii	idem #4
6	From the mobile application: download the video from the repository	ii, iii	Latency
7	From the UE: upload sensor data	ii, iii	Latency, Loss, Bit Rate
8	IoT Boxset: upload sensor data	ii, iii	Not applicable (Fully LAN-based communication)
9	From the mobile application and middleware: Monitor the *in_class* toggle and its effect on the received mobile sensors values	ii, iii	Latency

^a^ i) integration, (ii) validation and (iii) evaluation

We plan to run the series of tests cases in Table 3, with at least three iterations of 60 min each. The final evaluation will be carried out on fully-deployed mobile networks, we will leverage their Operation Support Systems (OSS) for tracking the selected QoS indicators [13].

Referring to the OSS results is the most reliable way for the selected indicators because these systems are developed to provide vital information about the activities in the networks. With the OSS values, we will be able to draw comparative charts and visually conclude the performances of the product on both networks, i.e., 4G and 5G.

## Conclusion

In this paper, we presented the design features of a 5G vertical product for blended learning environments, developed in the scope of a national 5G project. The paper's main objective was to research, develop, validate, integrate, and demonstrate both mechanisms and solutions that explore the functionality of future 5G networks for supporting human and IoT communication, targeting all the use cases envisioned for 5G. We have also proposed the design of innovative location-based service for blended learning environments.

In this scope, we presented the main product features and implementation tools for the proposed product. We adopted a bottom-up approach to design and develop the proposed product, to assess its behaviour when running OTT on both 4G and 5G networks set up by the project consortium. The next step will be to carry out the final integrated product validation, which will involve all the partners from the project.

The other future works consist of implementing the location-based service and assessing the network's ability to deal with the stringent real-time requirements.

## Supplementary Information

Below is the link to the electronic supplementary material.

Supplementary file1 (MP4 58999 KB)

## Data Availability

Not applicable.
